# American Medical Association

**Published:** 1848-07

**Authors:** 


*American Medical Association.*—The following are the officers and standing committees for the present year:—

*President.*—Dr. A. H. Stevens, of N. Y.

*Vice-presidents.*—Drs. John C. Warren, Mass., Samuel Jackson, Pa., W. M. Awl, Ohio, and Paul F. Eve, Ga.

*Secretaries.*—Drs. Alfred Stille, Pa., and H. I. Bowditch, Mass.

*Treasurer.*—Dr. Isaac Hays, Pa.

*Committee of Arrangements.*—Dr. Jacob Bigelow, *Chairman.*—Drs. E. Hale, Z. B. Adams, J. C. Dalton, John Ware, O. W. Holmes, and H. I. Bowditch, Boston.

*Committee on Medical Sciences.*—Dr. L. P. Yandell, Ky., *Chairman.*—Drs. S. M. Smith, Columbus, O., J. F. White, Cin., E. S. Carr, Vt., Samuel Jackson, (late of Northumberland,) Pa., G. S. Upshur, Va., and S. H. Harris. Tenn.

*Committee on Practical Medicine.*—Dr. D. F. Condie, Penn., *Chairman.*—Drs. W. W. Gerhard, Pa., M. Clymer, Phila., John Ware, Boston, Grafton Tyler, D. C., J. Fithian, N. J., and M. Z. Kreider, Ohio.

*Committee on Surgery.*—Dr. N. R. Smith, Md., *Chairman.*—Drs. H. F. Askew, Del., W. H. Baxley, Balt., J. Knight, Conn., J. Pancoast, Phila., H. H. McGuire, Va., and A. B. Shipman, Ind.

*Committee on Obstetrics.*—Dr. B. R. Welford, Va., *Chairman.*—Drs. J. F. Peebles, Va., Noble Young, D. C., Z. B. Adams, Mass., C. R. Gilman, N. Y., J. A. Eve., Ga., and R. Rouse, Ill.

*Committee on Medical Literature.*—Dr. J. P. Harrison, Ohio, *Chairman.*—Drs. G. Fries, Ohio, W. G. Edwards, Ill., W. M. Latta, Ind., O W. Holmes, Mass., R. S. Stewart, Md., and J. M. Thomas, D. C.

*Committee on Medical Education.*—Dr. F. Campbell Stewart, N. Y., *Chairman.*—Drs. John Watson., N. Y., J. M. Smith, N. Y., A. L. Pierson, Mass., S. H. Pennington, N. J., P. C. Gaillard, S. C., and D. Meeker, Ind.

*Committee on Publication.*—Dr. I. Hays, Phila., *Chairman.*—Drs. A. Stille, Phila., H. I. Bowditch, Mass., D. F. Condie, Phila., J. R. W. Dunbar, Md., B. F. Barker, Conn., and J. Jump, Del.

The next meeting of the Association will be held in Boston, on the first Tuesday in May.—*American Journal Medical Science.*

